# Characterization of Whey-Based Fermented Beverages Supplemented with Hydrolyzed Collagen: Antioxidant Activity and Bioavailability

**DOI:** 10.3390/foods9081106

**Published:** 2020-08-12

**Authors:** Arely León-López, Xóchitl Alejandra Pérez-Marroquín, Gieraldin Campos-Lozada, Rafael G. Campos-Montiel, Gabriel Aguirre-Álvarez

**Affiliations:** 1 Instituto de Ciencias Agropecuarias, Universidad Autónoma del Estado de Hidalgo, Avenida Universidad Kilometro 1, Tulancingo C.P. 43600, Hidalgo, Mexico; arlely@hotmail.com (A.L.-L.); ale.marr28@gmail.com (X.A.P.-M.); gieraldin.campos@gmail.com (G.C.-L.); rcampos@uaeh.edu.mx (R.G.C.-M.); 2 Uni-Collagen S.A. de C.V., Arnulfo González No. 203, El Paraíso, Tulancingo C.P. 43684, Hidalgo, Mexico

**Keywords:** milk whey, hydrolyzed collagen, antioxidant activity, bioavailability

## Abstract

In this study, the preparation of a milk whey-based beverage with the addition of different concentrations of hydrolyzed collagen (0.3%, 0.5%, 0.75%, and 1%) was carried out. The control was considered at a concentration of 0%. Physicochemical properties, viscosity, antioxidant activity, and microbiological parameters were evaluated. The 1% collagen treatment showed the highest protein content (9.75 ± 0.20 g/L), as well as radical inhibition for ATBS (48.30%) and DPPH (30.06%). There were no significant differences (*p* ≥ 0.05) in the fat and lactose parameters. However, the pH in the control treatment was lower compared to beverages treated with hydrolyzed collagen. Fourier transform-infrared spectroscopy showed spectra characteristic of lactose and collagen amides. The viscosity increased significantly as the concentration of hydrolyzed collagen increased. The addition of hydrolyzed collagen increased the bioavailability, nutritional value, and the antioxidant activity of the beverage. Hydrolyzed collagen acted as an antimicrobial agent, as there was no presence of microorganism pathogens observed in the treated beverages.

## 1. Introduction

The food industry has undergone constant changes due to high demands for food and the requirement of satisfying the nutritional needs of consumers. Functional foods are the main source of development and innovation in the food industry. These types of foods have been recognized as having physiological benefits beyond those of basic nutrition. Functional foods can be classified as whole, fortified, enriched, or enhanced foods or food compounds that have health benefits for the human body [1,2,3]. There is a wide range of functional foods, including baby foods, baked goods, cereals, dairy products, confectionery products, meat products, and beverages [4]. Functional beverages can be supplemented or enriched with functional ingredients, such as vitamins, minerals, bioactive peptides, probiotics/prebiotics, etc. [5]. Over 40% of functional foods are dairy products, and fermented beverages containing milk whey are the principal functional dairy beverage [6]. Milk whey is a translucent greenish yellow liquid that is produced during the coagulation of casein in cheese production. There are 2 types of whey: sweet whey (pH 5.9 to 6.5), which is obtained from rennet coagulation in hard and soft cheeses, and acid whey (pH 4.4 to 4.8), which is produced by acid-coagulated fresh milk [7]. The processing of 1 kg of cheese produces approximately 9 L of whey [8]. This whey is then discarded without treatment to public sewage systems, generating a critical pollution problem. Approximately only 50% of the whey produced globally is used to formulate products. The remainder is treated as waste. Whey has traditionally been dumped into surface water or fed to livestock. However, current environmental regulations and levies are forcing cheese makers to treat whey before disposal, and because of factory centralization, the cost of transporting whole whey for feed use has become prohibitive. Therefore, whey has become a liability and a great amount of research has been focused on converting this liability into an asset [9]. Fortunately, milk whey has good antimicrobial, antioxidant, and antiviral properties. The biological activity of whey is related to the composition and sequence of the amino acids obtained by lactic acid bacteria (LAB) during fermentation [10]. Whey possesses nutrients such as calcium, phosphorus, magnesium, and the vitamins riboflavin and thiamine; represents about 85–95% of milk volume; and retains up to 55% of its nutrients after processing [11,12]. The proteins present in milk whey are α-lacto albumin (13%), β-lactoglobulin (58%), immunoglobulins, and a low concentration of serum albumin [13,14]. The preparation of fermented beverages containing milk whey has the disadvantage of low solids and casein content, giving a watery consistency to the final products. However, the addition of fresh milk, condensed milk, milk powder, or some other additives to improve the textural and nutritional characteristics of the final products is common [15]. The first step to obtain hydrolyzed collagen (HC) is the denaturation of the native collagen identified for the separation of 3 α-chains. After that, the proteolytic enzyme action of alcalase, papain, and other enzymes is used to initiate hydrolysis. HC is a group of peptides with low molecular weight, around 6 KDa, that can be extracted from different sources (e.g., bovine, porcine, fish, ovine) and through different methods (alkaline methods using NaOH or NaHCO_3_, and acidic methods using acetic acid) [16]. Due to its low molecular weight, HC presents high biocompatibility, very easy degradation, and weak or no allergenicity [17]. In addition, HC is used as a functional ingredient within the food industry because of its antioxidant and antimicrobial activities. It helps to increase water holding, improving chemical and physical properties without modifying the sensorial properties in beverages and dairy products [18]. The objective of this research is to develop a beverage using milk whey and HC as functional ingredients to improve the nutritional, physicochemical properties, with antioxidant activity and bioavailability.

## 2. Materials and Methods

### 2.1. Milk Whey Characterization

The milk whey was donated by PROUNILAC Co. (Tulancingo, Hidalgo, Mexico). It was obtained as a byproduct from Oaxaca cheese processing by the coagulation of milk with organic acids. After slow pasteurization at 60 °C for 30 min, it was characterized for protein content [19], pH value [20], fat [21], and lactose [22].

### 2.2. Hydrolyzed Collagen

HC was obtained according to a previously determined methodology [23]. Native collagen was suspended in 1 M NaCO_3_ at a ratio of 1:4 (*w/v*). The pH was adjusted to 8 ± 0.2. The hydrolysis was treated under enzymatic conditions for 2 h at 70 °C. The molecular weight was reported to be around 5.62 KDa with 0 cP of viscosity.

### 2.3. Preparation of Beverage

First, 100 mL of functional beverage was prepared with milk whey and HC according to the methodology of Tirado-Armesto [24], with some modifications. Then, 10% (*w/v*) free lactose powder milk and 1% (*w/v*) sucrose was added to the milk whey and homogenized for 10 min at 3200 rpm using an Ultra-Turrax T-25 Digital (IKA; Wilmington, NC, USA) dispersing instrument. Later, milk whey was fermented by LAB: *Lactobacillus rhamnosus*, *Lactobacillus bulgaricus*, *Lactobacillus delbrueckii*, and *Streptococcus thermophilus* (0.001% *w/v*) (DANISCO, Paris, France) for 3 h at 37 ± 2 °C. The pH was tested during the fermentation process, until 6.0 ± 0.2 was reached. The fermentation process was stopped by decreasing the temperature to 4 °C. Different HC supplementation was added as follows: 0% (control), 0.3%, 0.5%, 0.75%, and 1% (*w/v*). The beverage was pasteurized at 60 °C for 30 min. Samples were stored at 4 °C for further analysis.

### 2.4. Protein Determination

The Bradford assay was used to determine the protein concentration [19]: 100 µL of the sample were mixed with 5 mL of Bradford reagent (Thermo Fisher Scientific; MA, USA) in a vortex for 2 min. The sample was stored in darkness for 5 min. The absorbance of the sample was read at 595 nm in a spectrophotometer (Jenway Genova, Model 6705, Bibby Scientific; Staffordshire, UK). A calibration curve of serum albumin at different concentrations was used.

### 2.5. Fat Determination

According to the Association of Official Analytical Chemists (AOAC) method [21], 11 mL of sample and 1 mL of isoamyl alcohol (Merck Millipore; Bedford, MA, USA) were added in a Gerber butyrometer. Then, sulfuric acid was added to cover the sample with gently shaking in a water bath at 65 °C for 10 min. The sample was centrifuged in a Gerber Centrifuge (Zelian, model Galatea 24; Buenos Aires, Argentina) at 12,000 rpm for 3 min followed for another 5 min in a water bath to complete the separation of fat. Readings were taken and expressed in mL of fat.

### 2.6. pH Value

The pH measurements were made in triplicate following the methodology described by AOAC [20] in a previously calibrated potentiometer (HANNA Instruments, model HI 2210; Limena, Italy).

### 2.7. Lactose Determination

The reaction process of carbohydrates by the anthrone method is an extremely sensitive and specific reaction that can be applied to all carbohydrates, whether or not they are reducers [25]. First, 1 mL of the sample was mixed with 2 mL of anthrone reagent (Sigma-Aldrich; St. Louis, MI, USA) into a water bath at 70 °C for 10 min. The sample was cooled to room temperature and a reading was taken using a spectrophotometer (Jenway Genova, Model 6705, Bibby Scientific; Staffordshire, UK) at a wavelength of 625 nm [19]. The equipment was calibrated with lactose solutions prepared at different concentrations.

### 2.8. Ash Determination

Following the AOAC [26] methodology, 3 g of sample were weighed into a porcelain crucible that was previously subjected to constant weight. The crucible was placed into a muffle furnace (BIOBASE, 1200c; Qingdao, China) at 550 °C for 5 h until complete calcination occurred. The crucible was cooled to room temperature, followed by the determination of the ash content by using Equation (1):

(1) $$\%\;\mathrm{Ash}=\frac{\left(C-A\right)}{B}\times 100,$$

where A is the crucible weight (g), B is the weight of the sample (g), and C is the weight of the crucible with ash (g).

### 2.9. Hydroxyproline Content

First, 4 g of the sample was mixed with 3.5 M sulfuric acid (30 mL) and incubated in an oven at 105 °C for 12 h. The volume was adjusted to 500 mL with distilled water followed by filtration. Then, 1 mL of oxidant solution (0.006 M chloramine T in 0.8 M citrate buffer, pH 6.0, Sigma-Aldrich; St. Louis, MI, USA) and 2 mL of the filtered sample were mixed in a reaction tube. The volume was adjusted to 100 mL and stirred for 30 min at 25 °C. Then, 2 mL of color reagent (10 g of dimethylamine benzaldehyde in 35 mL of 65% perchloric acid; Sigma-Aldrich; St. Louis, MI, USA) were added and stirred for 15 min at 60 °C [27]. The sample was read in a spectrophotometer (Jenway Genova, Bibby Scientific; Staffordshire, UK) at 558 nm. Equation (2) was used to calculated the hydroxyproline concentration:

(2) $$\%\;\mathrm{Hydorxyproline}=\frac{\left(\mathrm{Hydroxyproline}\;\mathrm{concentration}\;\mathrm{from}\;\mathrm{the}\;\mathrm{standard}\;\mathrm{curve}\right)\left(2.5\right)}{\left(\mathrm{sample}\;\mathrm{weight}\;\right)\left(\mathrm{volume}\;\mathrm{in}\;\mathrm{mL}\;\mathrm{to}\;\mathrm{adjust}\;\mathrm{to}\;100\;\mathrm{mL}\right)}.$$

### 2.10. Viscosity Analysis

Before the viscosity measurements, the samples were conditioned at 4 °C. A viscosimeter (Brookfield RTV; Middleboro, MA, USA) was used to determinate the sample viscosity by using spindle number 5 and a speed of 50 rpm. Results were expressed in centipoise (cP).

### 2.11. Fourier Transform-Infrared Spectroscopy

Fourier transform-infrared spectroscopy (FTIR) (Perkin Elmer; Boston, MA, USA) equipment was used to obtain absorption spectra within the range of 380 to 4000 cm^−1^ wavelengths at room temperature. Samples were placed in intimate contact with the diamond crystal by applying a loading pressure. Four scans with an average of 4 cm^−1^ resolution were represented in each sample. Automatic signals were collected in 3620 scans with a resolution of 1 cm^−1^. The spectrum of an empty cell was used as a background. Results were analyzed with Spectrum^TM^ 10 software (Perkin Elmer, Boston, MA, USA).

### 2.12. Differential Scanning Calorimetry

Differential scanning calorimetry equipment series Q 2000 with an intracooler RCS90 (TA Instruments; New Castle, Delaware. USA) was calibrated with indium (T_m_, onset = 156.68 °C, ∆H = 28.45 J/g). Then, 1.5 ± 0.1 mg of the sample with 0% db water content was packed and hermetically sealed in a 50-mL stainless steel pan. An empty pan was used as a reference. Both heating and cooling scan rates were performed at 10 °C/min. Two heating scans were performed from 25 to 120 °C. TA 2000 analysis software (TA Instruments; New Castle, DE, USA) based on the endothermic changes registered in the thermogram was used to determine the melting temperature (T_m_) and enthalpy (∆H).

### 2.13. Antioxidant Activity

The ABTS (2,2′-Azino-bis(3-ethylbenzothiazoline-6-sulfonic acid)) radical solution was prepared according to Re et al.’s method [28]. First, 2.45 mM potassium persulfate were mixed with 7 mM of ABTS (Sigma-Aldrich; St. Louis, MI, USA). The mixture was stirred at room temperature for 16 h in the darkness. The ABTS solution was stabilized to 0.70 ± 0.02 at 734 nm using ethanol. Then, 2 mL of the sample and 1 mL of stabilized radical ABTS solution were mixed, and after 6 min, the sample was read at 734 nm in a spectrophotometer (Jenway Genova, Model 6705, Bibby Scientific; Staffordshire, UK).

A similar methodology was applied to samples for DPPH (2,2-diphenyl-1-picrylhydrazyl) antioxidant activity [29]. In this case, 0.5 mL of the sample was mixed with 2.5 mL of 6.1 × 10^−5^ M of DPPH radical inhibitor (Sigma-Aldrich; St. Louis, MI, USA). The mixture was stored in darkness for 30 min and then read in a spectrophotometer (Jenway Genova, Model 6705, Bibby Scientific; Staffordshire, UK) at 515 nm. The antioxidant activity of both ABTS and DPPH radical scavengers were calculated according to Equation (3):

(3) $$\%\;\mathrm{Inhibition}=\frac{\mathrm{Ia}-\mathrm{Fa}\;}{\mathrm{Ia}}\times 100,$$

where Ia is the initial absorbance and Fa is the final absorbance.

### 2.14. Microbiological Analysis

Determination of *Salmonella* and *E. coli* were followed according to Mexican legislation (NOM-210-SSA-2014) as an indicator of microorganisms of contamination, using Salmonella Shigella Agar (Becton Dickinson; Heidelberg, Germany) and Eosin Methylene Blue Agar (Becton Dickinson; Heidelberg, Germany), respectively. For the determination of molds and yeasts in food (Sabouraud Dextrose Agar, Becton Dickinson; Heidelberg, Germany), the methodology of NOM-11-SSA-1994 was followed. In addition, NOM-092-SSA-1994 was followed for the aerobic bacteria count (aerobic mesophiles) by using Standard Methods Agar (DIBICO; Cuautitlan Izcalli, Mexico). The samples were incubated in petri dishes (90 × 15 mm) for 24 h in an oven (FELISA model FE-131; Zapopan, Jalisco. Mexico) at 37 ± 2 °C. The results were expressed as the presence or absence of microorganisms that are indicative of an efficient pasteurization process.

### 2.15. In Vitro Bioavailability of Hydrolyzed Collagen in Beverages

The gastric simulation methodology of Bilek and Bayram [30] was used with some modifications. First, 50 mL of the beverage was mixed with 6.5 mg of pepsin (15,750 units; Sigma-Aldrich; Munich, Germany). The pH of the sample-pepsin solution was adjusted to 2.0 with 1 M HCl and incubated at 37 °C for 2 h. Subsequently, 10 mL were taken and transferred to a dialysis membrane (2 KDa) and mixed with 2.5 mL of a bile acid-pancreatin (Sigma-Aldrich; Munich, Germany). The pH was adjusted to 7.5 with 0.5 M NaHCO_3_. Along with this experiment, a buffer solution was prepared in a centrifuge tube with 10 mL of deionized water, the same amount of 0.5 M NaHCO_3_, and adjusted to pH 5.0 with 1 M HCl. The dialysis membrane with the sample-pepsin solution was placed inside the centrifuge tube with a buffer and incubated at 37 °C for 2 h. The sample inside the membrane was subjected to hydroxyproline analysis to determine the amount of collagen present after gastric simulation. The percentage of bioavailability was calculated by using Equation (4):

(4) $$\%\;\mathrm{Bioavailability}=\frac{\mathrm{Collagen}\;\mathrm{in}\;\mathrm{dialysate}\;\left(g\right)\;}{\mathrm{Collagen}\;\mathrm{in}\;\mathrm{sample}}\times 100.$$

### 2.16. Statistical Analysis

A randomized design experiment was applied. Three replicates were considered in this experiment. Analysis of variance (ANOVA) and Tukey tests (*p* ≤ 0.05) were also used. Data were processed with SPSS software v25 (SPSS Inc.; Chicago, IL, USA).

## 3. Results and Discussion

### 3.1. Acid Milk Whey Chemical Characterization

The characteristics of the raw material were as follows: pH of 4.4 ± 0.2, 0.4 ± 0.4 g/L of total solids, 2.4 ± 0.4 g/L of total proteins, 29.3 ± 0.1 g/L of lactose, and 0.4 ± 0.1 g/L of fat. These results appeared above the average general composition of whey in terms of protein and lactose concentration [31]. Whey from semi-fat quark cheese production showed a higher solids concentration and pH, but a lower protein presence [32]. Whey from Ras cheese production resulted in higher fat and solids concentrations, but lower protein and lactose concentrations [33]. The composition of the milk whey depends on the source of the milk, type of cheese, and the cheese processing method [34,35,36,37]. The variability in the chemical composition might be due to the fact that the cheese production is a non-standardized process, so depending on the production methodology, the final characteristics of the whey will be affected [38,39].

### 3.2. Physicochemical Characterization of The Functional Beverage

Table 1 shows the functional beverage characterization. There was no significant difference (*p* ≤ 0.05) between the resulting pH values and the treatments with HC (~7.0). However, the control treatment (0%) showed a lower pH value (5.1) compared to the others. There was a higher resulting pH in samples with HC because the hydrolysis of collagen was carried out in an alkaline medium (NaHCO_3_). Beverages based on milk whey fermentation present some advantages: a decrease in lactose content, partial hydrolysis of whey protein, increase in the production of lactic acid, and production of aroma compounds that improve sensory characteristics [14,40]. However, there is a disadvantage in its low total solids content when compared to milk. Therefore, the addition of HC, sucrose, and milk powder could fix this problem. The acid lactic bacteria used for fermentation in this experiment (*L. rhamnosus*, *L. bulgaricus*, *L. delbrueckii*, and *S. thermophilus*) belonged to a group of lactose consuming microorganisms. These types of bacteria can improve the odor and flavor in fermented beverages [41]. The beverage protein concentration was 9.75 ± 0.20 g/L in the treatment with the highest collagen concentration (1%). It presented a significant difference (*p* ≤ 0.05) compared to the treatments with the lowest collagen concentration and the control. These results suggest that the addition of HC could increase the nutritional value of the beverage. Hailu et al. [42] conducted a study based on whey beverages that presented high nutritional value and an ideal source of energy and nutrients. The ash content in the functional beverage provided an estimate of the minerals present in the sample. Minerals were found in ash as oxides, sulfates, phosphates, nitrates, chlorides, and other halides. The predominant minerals in milk whey are sodium and potassium [13,43]. The fat content in the functional beverage did not show a significant difference (*p* ≥ 0.05) between the treatments; the values were between 0.20 ± 0.10 and 0.23 ± 0.06 g/L. These results are similar to those reported in previous works [44,45] based on the preparation of beverages with fermented milk whey and low fat content. The same behavior was observed for lactose parameters with no significant differences (*p* ≥ 0.05) no matter what the amount of HC is.

### 3.3. Viscosity Determination of the Whey Beverage

**Figure 1:**
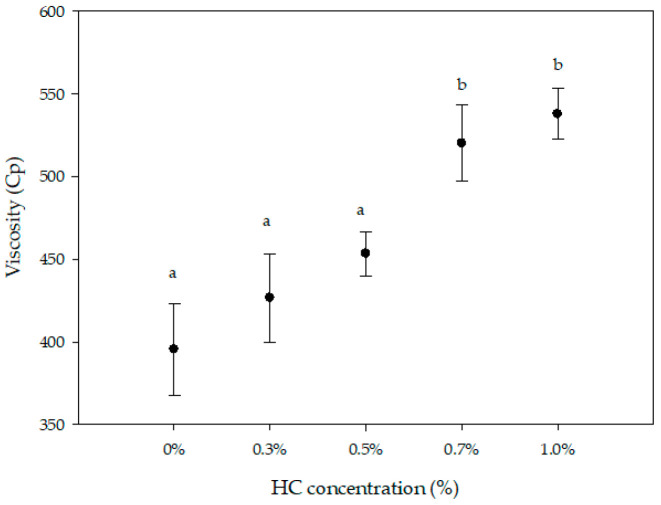
Beverage viscosity with different concentrations of hydrolyzed collagen (HC). Values are means ± SD based on 3 observations. Means with different lowercase letters differ (*p* ≤ 0.05).

Figure 1 showed significant difference (*p* ≤ 0.05) in viscosity between the highest concentration of HC addition (1%) and the lowest HC addition (0.3%). Viscosity increased from 395.55 ± 27.75 Cp to 537.7 ± 15.39 Cp, respectively. In dairy beverages, viscosity is an important characteristic for the consumer’s acceptance and it depends on the solids content; type and concentration of additives; and fermentation conditions like time, temperature, and the LAB used [46]. The higher the HC concentration, the higher the viscosity observed in the beverages. HC is used in food to improve the stability because it is able to reduce the surface tension at the liquid interface by increasing the viscosity of the aqueous phase. In addition, it has been reported that HC presented greater water retention capacity, reducing syneresis and sedimentation in the beverage [47,48,49]. Previous works also presented a change in the viscosity in whey beverages by incorporating some other components, such as açaí pulp, HC, and sweet potato flour [49,50]. Additionally, the addition of soy protein in fermented whey beverages raised the viscosity [51]. Cassava starch is also a common option to obtain the desired viscosity and stability characteristics to improve the texture and sensory properties of whey beverages [52]. Dairy products are complex in composition and structures, showing differences in viscosity. The addition of some natural ingredients can help to achieve physicochemical properties like viscosity.

### 3.4. Fourier Transform-Infrared Spectroscopy

**Figure 2:**
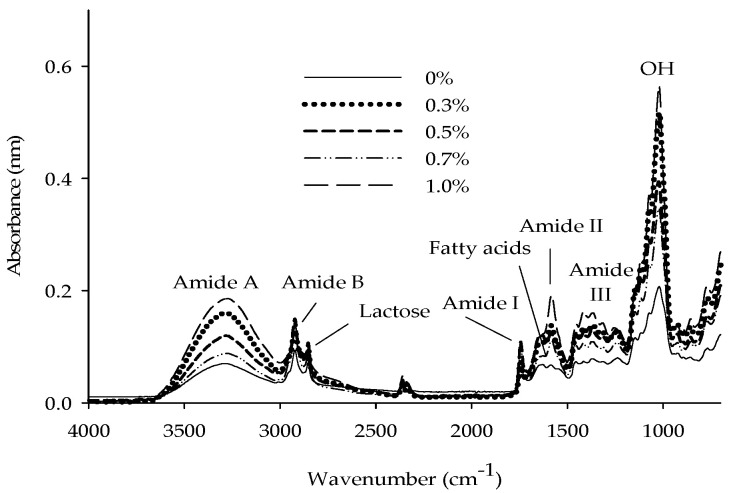
Fourier transform-infrared spectra of the functional whey beverage with different concentrations of hydrolyzed collagen.

When using the FTIR technique, the samples do not require pretreatment or the use of any organic solvents, which reduces the environmental damage caused by toxic waste. The FTIR technique is based on natural vibrational frequencies of the chemical bonds present in the molecules [53]. As illustrated in Figure 2, the peaks between 1644 and 1649 cm^−1^ are representative of the fatty acids present in dairy products, and 1080 cm^−1^ is representative of lactose [54,55]. The characteristic peaks for collagen were observed at 3295 cm^−1^, which is related to the tension vibrations of the NH group (Amide A), and 2946 cm^−1^, which is related to the asymmetric stretching of the CH_2_ groups (Amide B). Additionally, the peak at 1641 cm^−1^ is representative of Amide I, which is correlated with the α-helix chains and is used specifically to analyze the secondary structure of collagen. Amide II (1548 cm^−1^) and Amide III (1248 cm^−1^) were mainly associated with intermolecular interactions, representing the stretching vibrations of the CN group and the deformation of the NH group of the amide bonds, respectively. These peaks were observed in a previous work where HC was extracted [23]. The increase in the amplitude in the region of 3000 to 3500 cm^−1^ was also attributed to hydrogen bonds, due to the presence of the molecular chains interacting by inter- and intra-molecular hydrogen bonds [56]. The peak around 1000 cm^−1^ can be related to the presence of lactose and OH groups in the amino acid hydroxyproline, which is the main amino acid present in HC. The increment in amplitude of this peak is due to increased CH concentrations in the beverage [23,54]. Previous works also found Amide I and Amide II in the same region and these peaks were related to the amount of the protein present in the sample [57,58].

### 3.5. Thermal Properties of Whey Beverage

Table 2 did not present significant difference (*p* ≥ 0.05) in Tm between the different treatments and the control. However, significant differences (*p* ≤ 0.05) were observed between the control treatment (0%) and the treatments with HC. The presence of HC in the beverage not only favors the thermal stability, but also helps to increase pH (Table 1). Fitzsimons et al. [59] showed the relation between the differential scanning calorimetry and the protein content increments, presence of salt, and pH in whey products. Heating treatment causes the denaturation of proteins and thus causes changes in the functional and structural characteristics of proteins [60]. Changes in pH could be affected by the thermal properties in whey products [61,62]. The presence of other components, such as HC, sucrose, and lactose-free milk powder, can also modify the composition of the beverage. Joyce et al. [63] reported similar results in milk formulas with different concentrations of whey protein: the product with a higher protein content presented a higher denaturation temperature.

### 3.6. Antioxidant Activity by ABTS and DPPH Radical Inhibition

**Figure 3:**
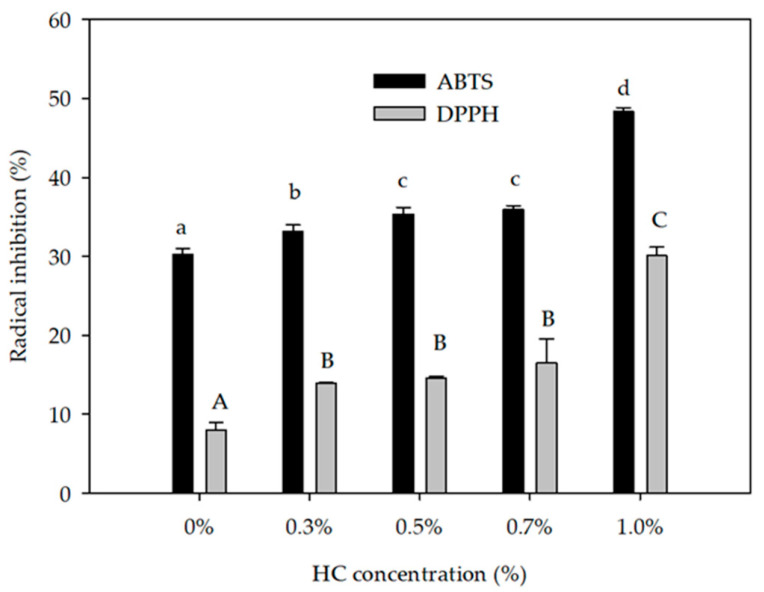
Antioxidant activity by ABTS (2,2′-Azino-bis(3-ethylbenzothiazoline-6-sulfonic acid)) and DPPH (2,2 diphenyl-1-picrylhydrazyl) radical inhibition of the whey beverage with different concentrations of added hydrolyzed collagen (HC). Values are means ± SD based on 3 observations. Means within the same category (i.e., ABTS or DPPH) with different letters differ (*p* ≤ 0.05).

Figure 3 shows that the highest radical inhibition for both ABTS and DPPH resulted in treatment with the highest HC (1%) with values of 48.30 ± 0.52% and 30.06 ± 1.09%, respectively. These values were different (*p* ≤ 0.05) compared to the 0% (control) concentration. This antioxidant activity is related to the hydrophobic and aromatic amino acids, which can stabilize electron deficient radicals by donating protons [64]. However, the increase of antioxidant activity could be related to the addition of HC; when the HC concentration is increased, the radical inhibition is higher. The strong hydrolysis of collagen increased the concentration of isoleucine and methionine along the reduction of molecular weight and increased antioxidant activity [23]. Additionally, previous works have found that HC antioxidant activity is related to amino acid composition (tyrosine, histidine, and methionine) and low molecular weight. Isoleucine and methionine can be electron donors or hydrogens contributing to the increase of radical scavenging [65,66]. In beverages, the presence of bioactive peptides from both HC and milk whey not only contribute to their physicochemical stability, but also in the opportunity that these substances reach the consumer and promote their health [13]. Other works in the development of fermented whey beverages presented low ABTS radical inhibition compared to the inhibition obtained in this research [67,68]. In addition, DPPH radical inhibition was lower in beverages with orange, soursop, and carrot juice [69,70,71].

### 3.7. Microbiological Analysis

The beverage was pasteurized for 30 min at 60 °C. The main objective of this thermal treatment was the inhibition or elimination of microorganisms that can affect the quality of the food product. Thermal treatment was successful since no contamination by microorganisms were present (day 0). Additionally, the presence of the raw materials, such as whey and HC, could have a positive effect in inhibiting the presence of microorganisms. The antimicrobial property of the HC is related to the low molecular weight between 3–6 kDa and the presence of free amino acids [13,72]. In addition, milk whey is a good source of biological proteins, peptides, minerals, and vitamins, helping with the growth of LAB, and producing inhibitory metabolites that are antagonistic to pathogens [73]. The proteins present in milk whey, such as α-lacto albumin, β-lactoglobulin, or lactoferrin, are physiologically active and present antimicrobial and antiviral activities. However, the beverage with 0% HC after 15 and 30 days of storage at 4 °C was positive for yeast and mold. The treatments with HC did not show the presence of *Salmonella*, *E. coli*, molds, yeast, or anaerobic mesophiles. The HC, as a functional ingredient, could act as a natural antimicrobial to help extend the shelf-life of food. Previous works showed the presence of aerobic mesophiles, *E. coli*, mold, and yeast in an orange juice whey beverage [64], whey-based sports beverages [74], and a whey beverage with lutein [75]. However, when HC was added as a functional ingredient to different types of foods like soup [76], sausages [77], orange and apple juice with HC, dairy beverages [49], and fermented dairy beverages [48], lower or no presence of pathogenic microorganisms, such as *Salmonella* and aerobic mesophiles, were shown.

### 3.8. In Vitro Bioavailability

**Figure 4:**
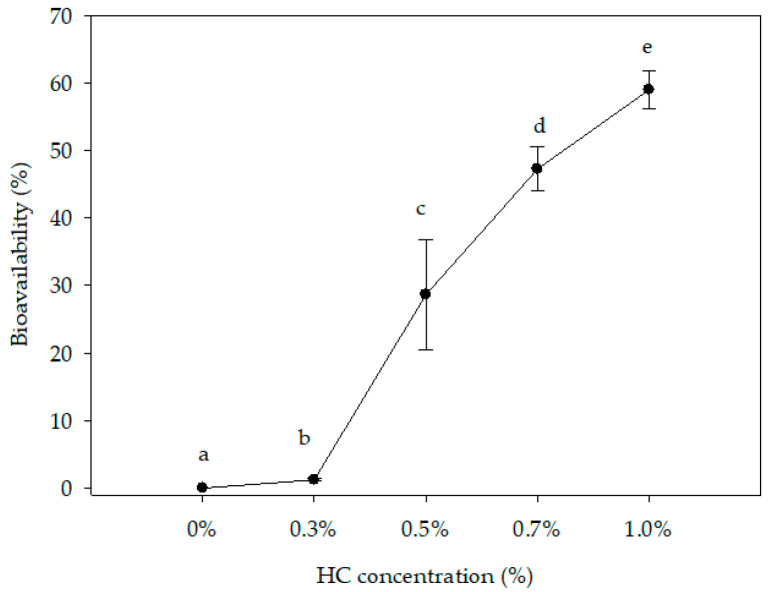
In vitro bioavailability of the hydrolyzed collagen (HC) present in the whey beverage. Values are means ± SD based on 3 observations. Means with different lowercase letters differ (*p* ≤ 0.05).

Bioavailability describes the amount of nutrients or functional ingredients that are actually absorbed, distributed to the tissues, metabolized, and eventually excreted by the body [78,79]. Figure 4 shows the in vitro bioavailability of HC present in the whey beverages. All the treatments resulted with significant differences (*p* ≤ 0.05). Beverages with a 0.3% addition of HC showed the lowest rate of absorption at 1.22 ± 0.15%. When HC was increased, the rate of absorption showed the same behavior up to 58.95 ± 2.72% for the 1.0% treatment. HC was added as a functional ingredient into the whey beverage to modify the physicochemical properties to increase the functional properties, such as the antimicrobial and antioxidant properties already mentioned. The advantage of the HC used in this experiment is related to its low molecular weight (5.79 ± 0.30 KDa) and hydroxyproline concentration of about 17.85 ± 1.22 mg/L [20]. Peptides with low molecular weight, such as HC, are more bioavailable compared to native proteins [80,81,82]. When collagen is consumed in the form of a native protein, it needs to undergo degradation by gastric and pancreatic protease action, to become small peptides or free amino acids. Subsequently, peptides could be transported by the intestine in the form of free amino acids and di- or tripeptides, which results in poor assimilation. However, HC plays an important role because collagen in its hydrolyzed form can be easily assimilated by the body due to previous digestion with enzymes [83,84,85,86]. In addition, the presence of hydroxyproline promotes the major assimilation of HC because this amino acid along with proline can be absorbed in the gastrointestinal tract after ingestion [82]. Similar results were shown in a juice beverage with HC: the higher the HC concentration in the beverage, the higher the bioavailability was observed [30].

## 4. Conclusions

From this study, it can be concluded that the presence of both HC and milk whey in a functional beverage helped to increase the nutritional value of the beverage due to the high protein concentration and no changes in the fat and lactose concentrations. HC played the role of an antioxidant ingredient and prevented the presence of some pathogenic microorganisms. Additionally, the in vitro bioavailability of HC demonstrated high rates of absorption due to its low molecular weight. These results could be of great importance for their application in the development of beverages oriented towards athletes and elderly people.

## Figures and Tables

**Table 1:** Functional beverage physicochemical characterization.

Hydrolyzed Collagen Concentration (%)	pH	Ash (%)	Fat (g/L)	Lactose (g/L)	Protein (g/L)
0	5.10 ± 0.03 ^b^	0.82 ± 0.11 ^c^	0.20 ± 0.10 ^a^	27.09 ± 10.63 ^a^	9.13 ± 0.09 ^c^
0.3	7.07 ± 0.01 ^a^	0.83 ± 0.11 ^bc^	0.23 ± 0.06 ^a^	18.65 ± 8.60 ^a^	9.35 ± 0.10 ^b^
0.5	7.03 ± 0.03 ^a^	1.14 ± 0.02 ^ab^	0.27 ± 0.06 ^a^	20.10 ± 3.34 ^a^	9.45 ± 0.07 ^b^
0.7	7.36 ± 0.03 ^a^	1.03 ± 0.01 ^a^	0.27 ± 0.06 ^a^	15.12 ± 3.62 ^a^	9.48 ± 0.11 ^b^
1.0	7.39 ± 0.03 ^a^	1.19 ± 0.06 ^a^	0.23 ± 0.06 ^a^	28.26 ± 6.31 ^a^	9.75 ± 0.20 ^a^

Values are means ± SD based on 3 observations. Means with different lowercase letters differ (*p* ≤ 0.05).

**Table 2:** Enthalpy (∆H) and melting temperature (T_m_) of fermented whey beverages following thermal treatment at 60 °C for 30 min.

Hydrolyzed Collagen Concentration (%)	∆H (J/g)	T_m_ (°C)
0	94.81 ± 6.01 ^a^	163.504 ± 5.16 ^a^
0.3	12.77 ± 4.23 ^b^	158.88 ± 5.84 ^a^
0.5	8.80 ± 6.07 ^b^	147.03 ± 4.43 ^a^
0.7	11.53 ± 2.26 ^b^	165.612 ± 2.75 ^a^
1.0	14.818 ± 3.37 ^b^	161.4 ± 5.90 ^a^

Values are means ± SD based on 3 observations. Means with different lowercase letters differ (*p* ≤ 0.05).

## Abbreviations

ABTS	2,2′-Azino-bis (3-ethylbenzothiazoline-6-sulfonic acid)
ANOVA	analysis of variance
DPPH	2,2-diphenyl-1-picrylhydrazyl
FTIR	Fourier transform-infrared spectroscopy
HC	hydrolyzed collagen
LAB-	lactic acid bacteria
